# Supplementary figures and images for: Variability in Bacteriophage and Antibiotic Sensitivity in Serial Pseudomonas aeruginosa Isolates from Cystic Fibrosis Airway Cultures over 12 Months
Source: Microorganisms. 2021 Mar 22;9(3):660. doi: 10.3390/microorganisms9030660 (PMC8004851; doi:10.3390/microorganisms9030660)

## Slide 1
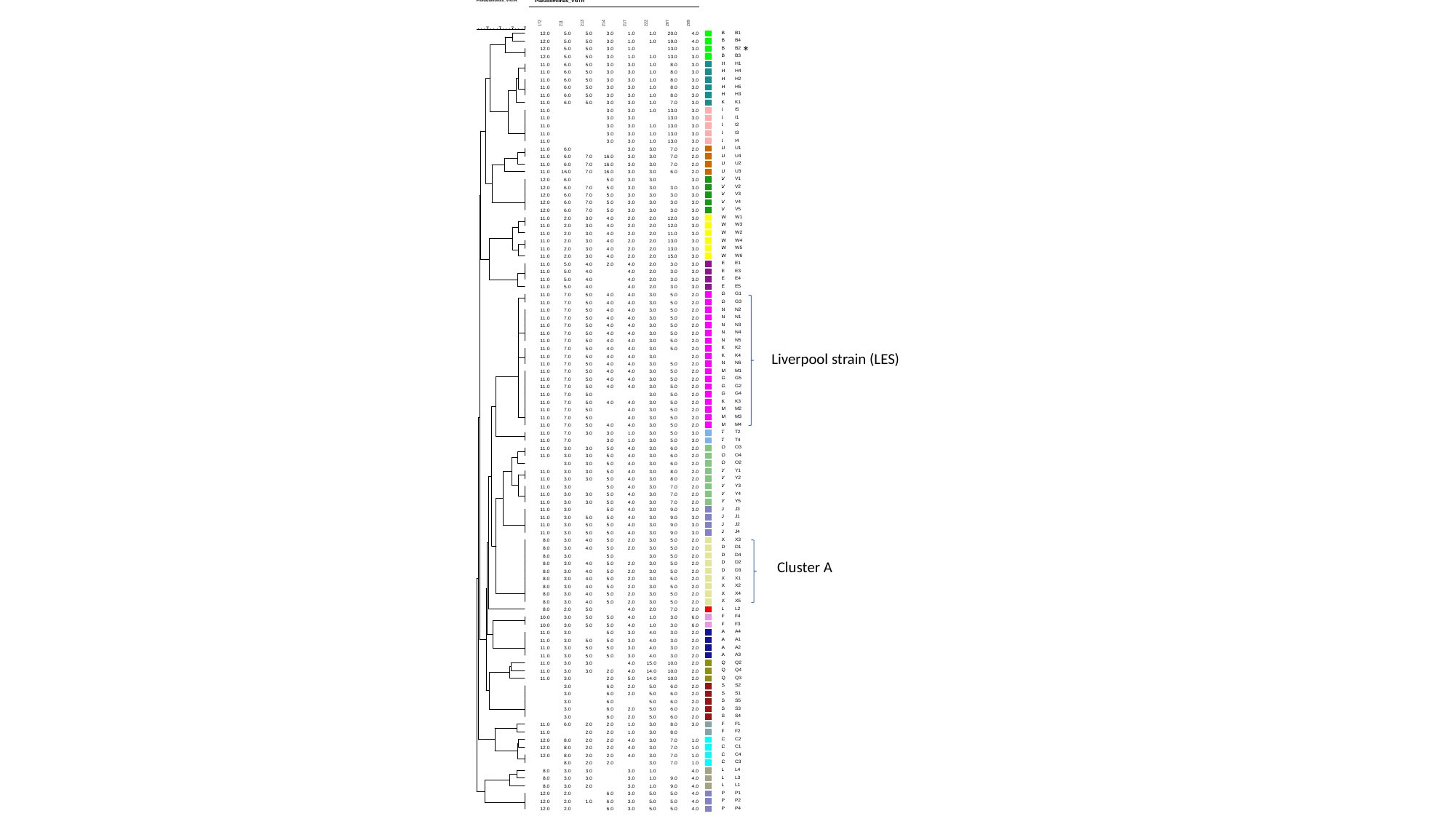

*
Liverpool strain (LES)
Cluster A

Supplement: Supplementary file 1 [file microorganisms-09-00660-s001.pptx]
